# Supplementary material for: SingleCellSignalR: inference of intercellular networks from single-cell transcriptomics
Source: Nucleic Acids Res. 2020 Mar 20;48(10):e55. doi: 10.1093/nar/gkaa183 (PMC7261168; doi:10.1093/nar/gkaa183)
Supplement: gkaa183_Supplemental_Files [file gkaa183_supplemental_files.zip › SCSignalR-supplementary.pdf]

SUPPLEMENTAL MATERIAL

SingleCellSignalR: Inference of intracellular networks from single cell transcriptomics

Simon Cabello-Aguilar[1], Fabien Kon Sun Tack[1], Mélissa Alame[1,2], Caroline Fau[1], Matthieu Lacroix[1], Jacques Colinge[1,*]

[1]Institut de Recherche en Cancérologie de Montpellier, Inserm, F-34298 Montpellier, France ; Institut régional du Cancer Montpellier, F-34298 Montpellier, France ; Université de Montpellier, F-34090 Montpellier, France

[2]Département d'Hématologie biologique, CHU Montpellier, Hôpital Saint Eloi, F-34090 Montpellier, France

*Correspondence:

Prof. Jacques Colinge
IRCM, Inserm U1194
208 rue des Apothicaires
34298 Montpellier cedex 5
France
Tel : +33 (0)467 612392
FAX : +33 (0)467 613787
E-mail : jacques.colinge@inserm.fr

Supplementary Figures & Tables

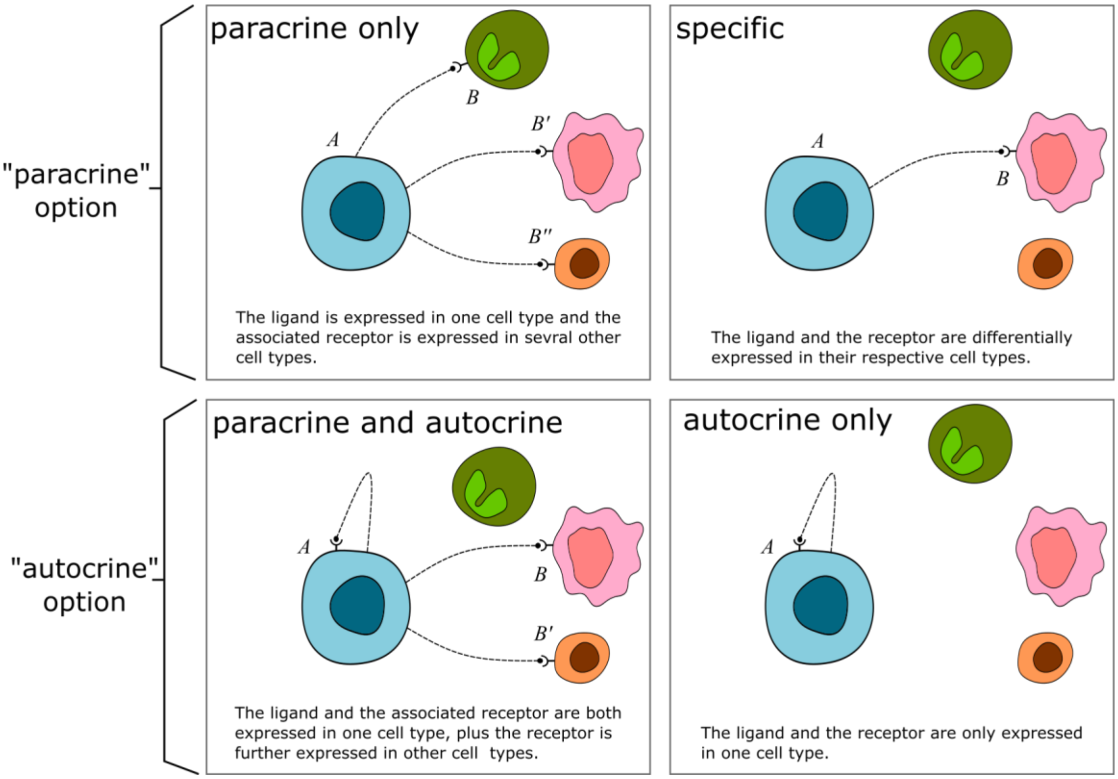

Supplementary Figure 1. Different types of cellular interactions between cell populations A and B can be inferred by SingleCellSignalR depending on the chosen options. The *specific* case is a particular case of the paracrine interaction.

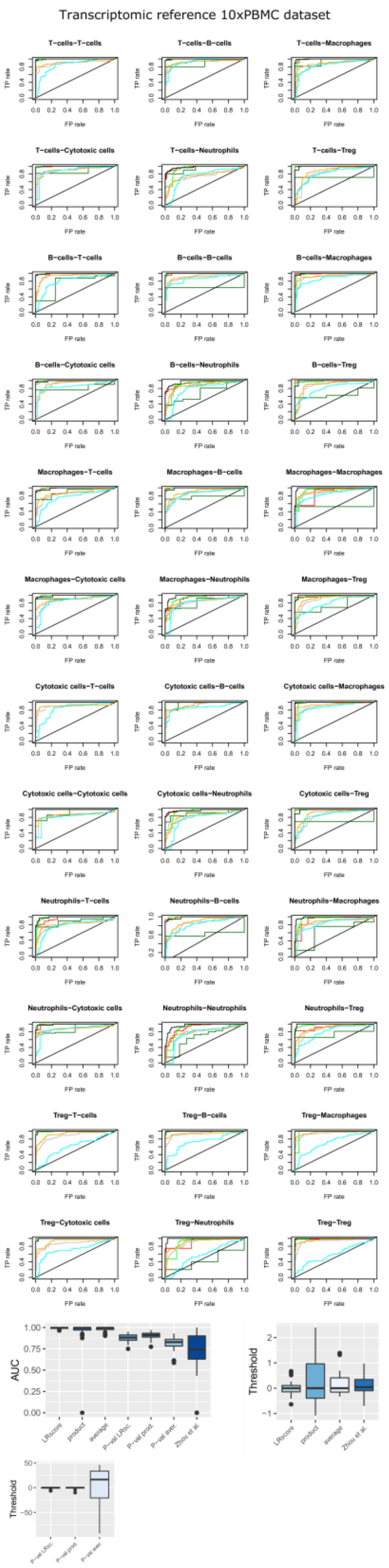

Supplementary Figure 2. ROC curves of LR pairs across cell populations of the 10xPBMC data set (1) with respect to the transcriptomic reference (2).

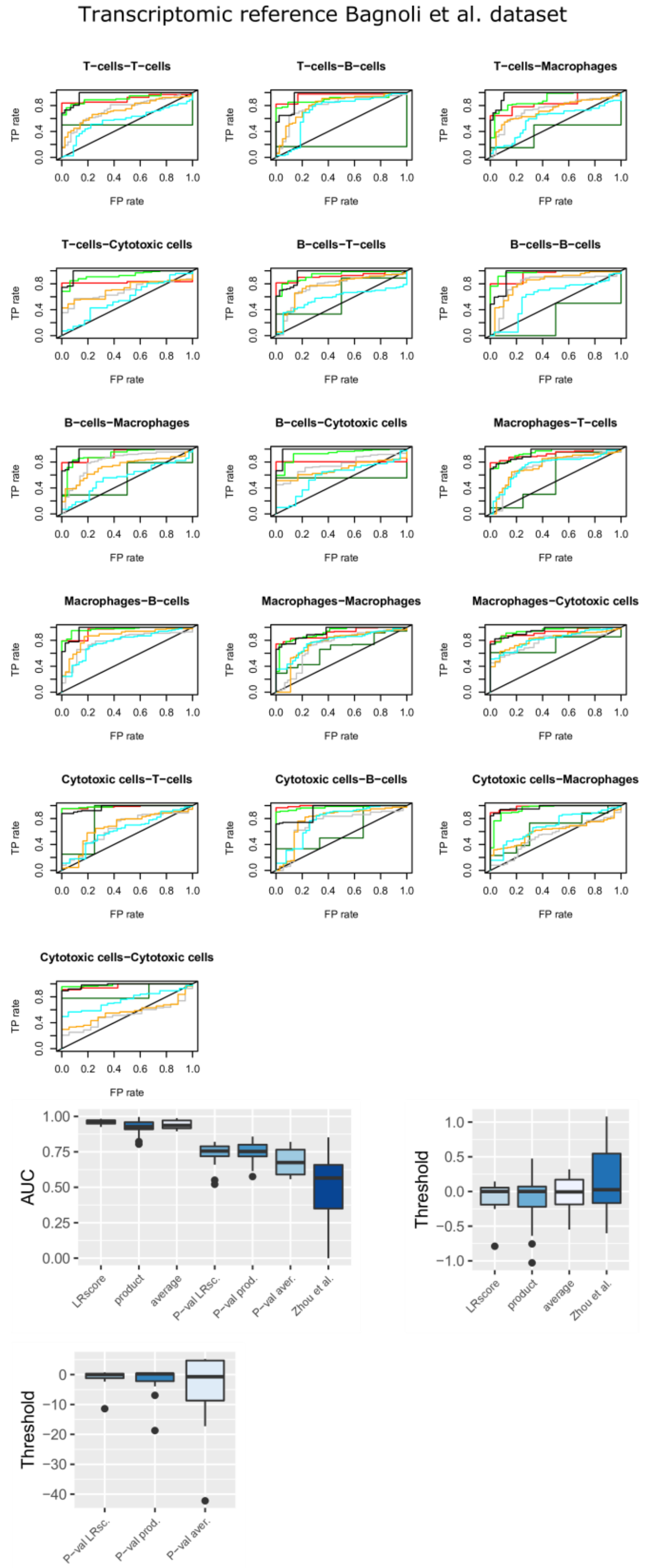

Supplementary Figure 3. ROC curves of LR pairs across cell populations of the PBMC data set (3) with respect to the transcriptomic reference (2).

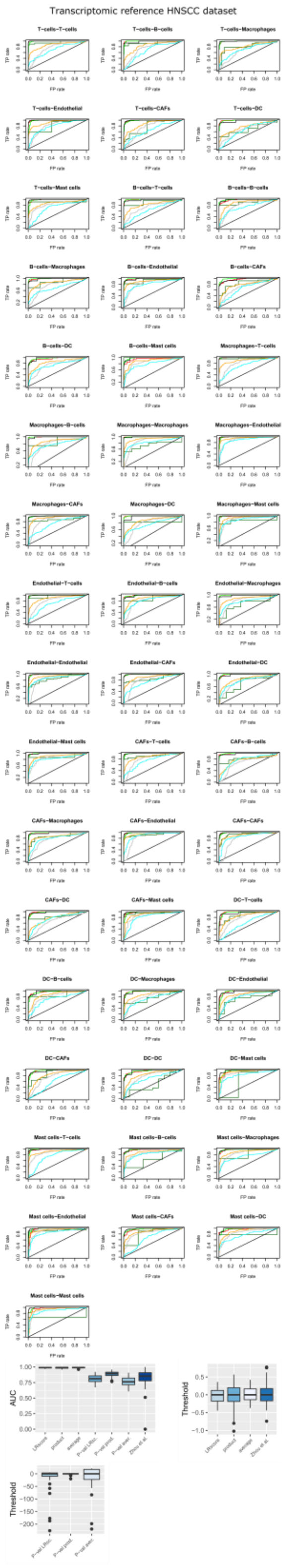

Supplementary Figure 4. ROC curves of LR pairs across cell populations of the HNSCC data set (4) with respect to the transcriptomic reference (2).

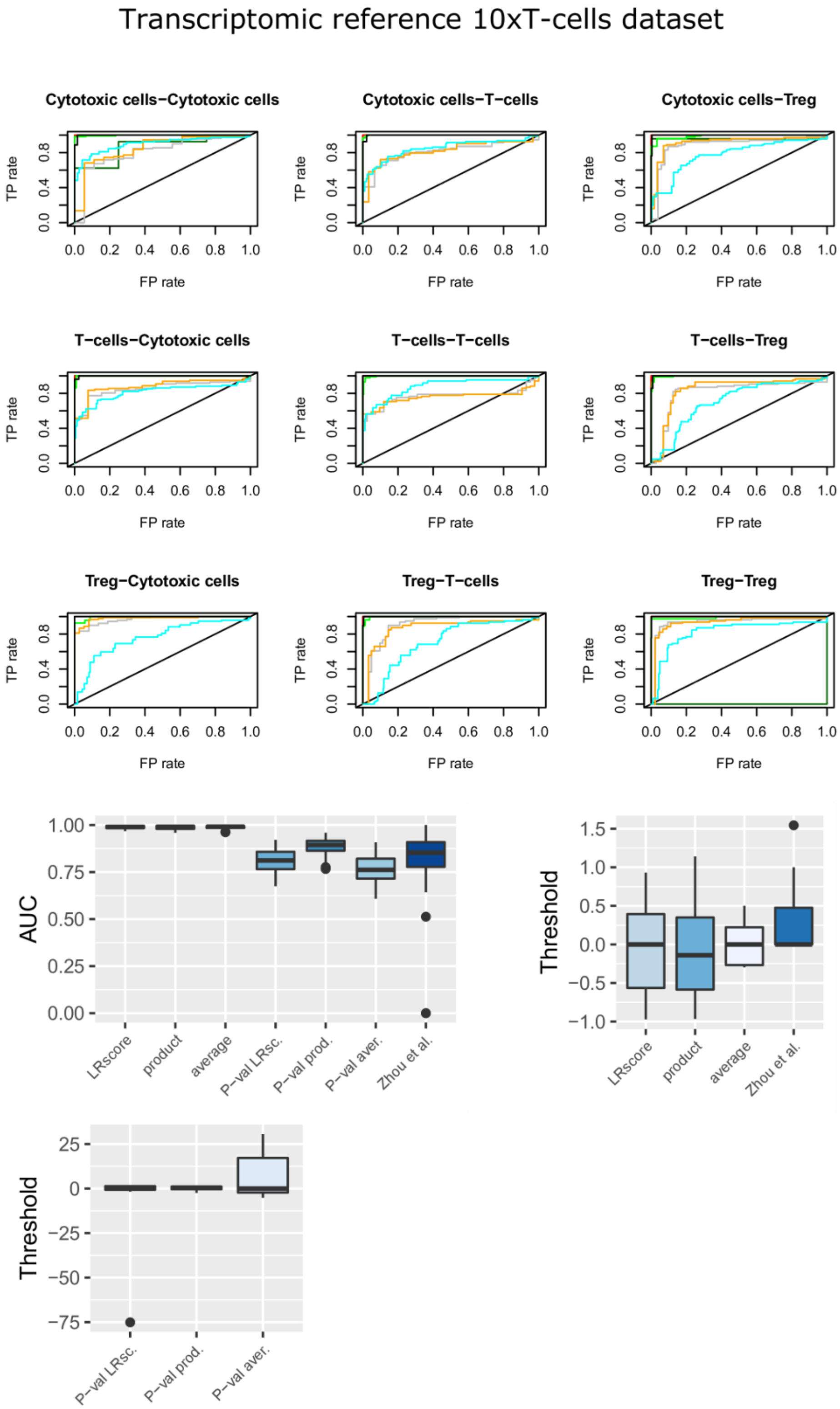

Supplementary Figure 5. ROC curves of LR pairs across cell populations of the 10xT data set (5) with respect to the transcriptomic reference (2).

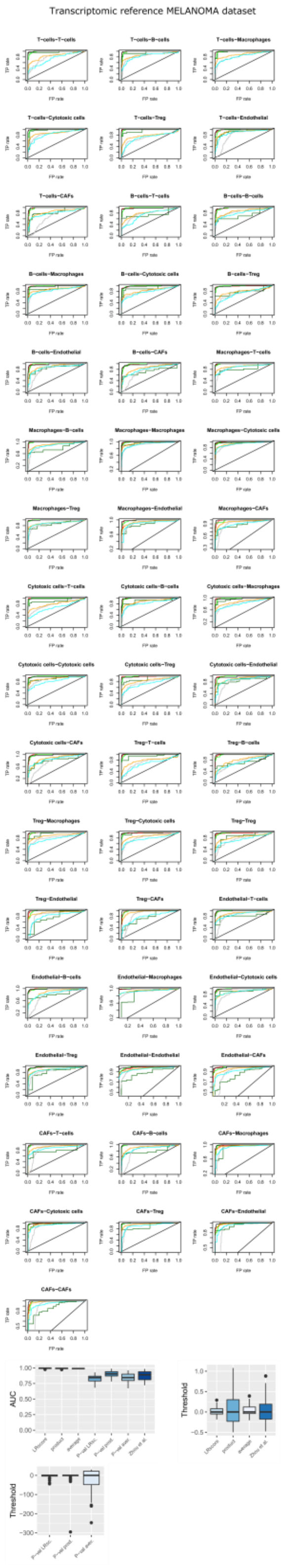

Supplementary Figure 6. ROC curves of LR pairs across cell populations of the MELANOMA data set (6) with respect to the transcriptomic reference (2).

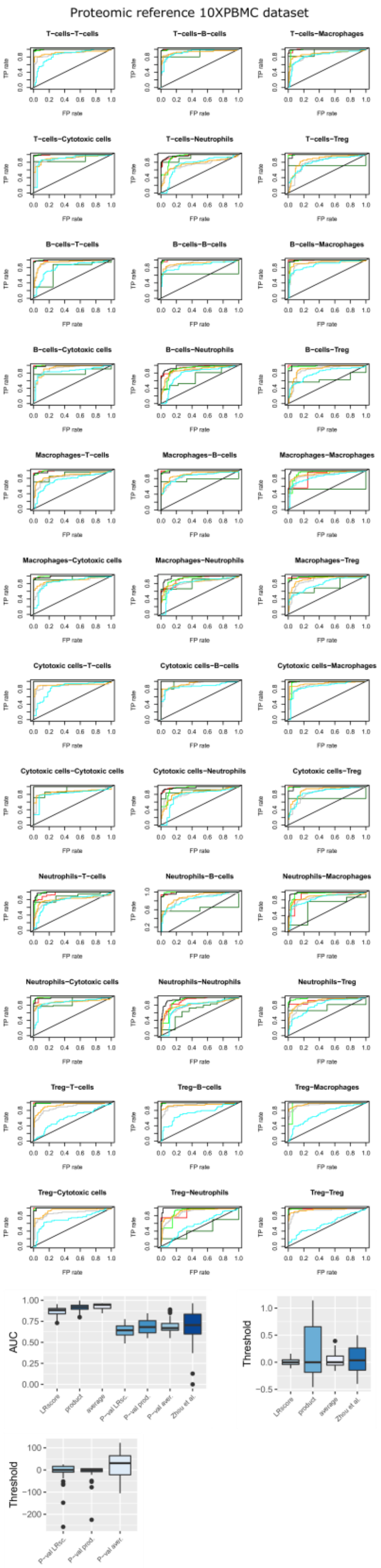

Supplementary Figure 7. ROC curves of LR pairs across cell populations of the 10xPBMC data set (1) with respect to the proteomic reference (7).

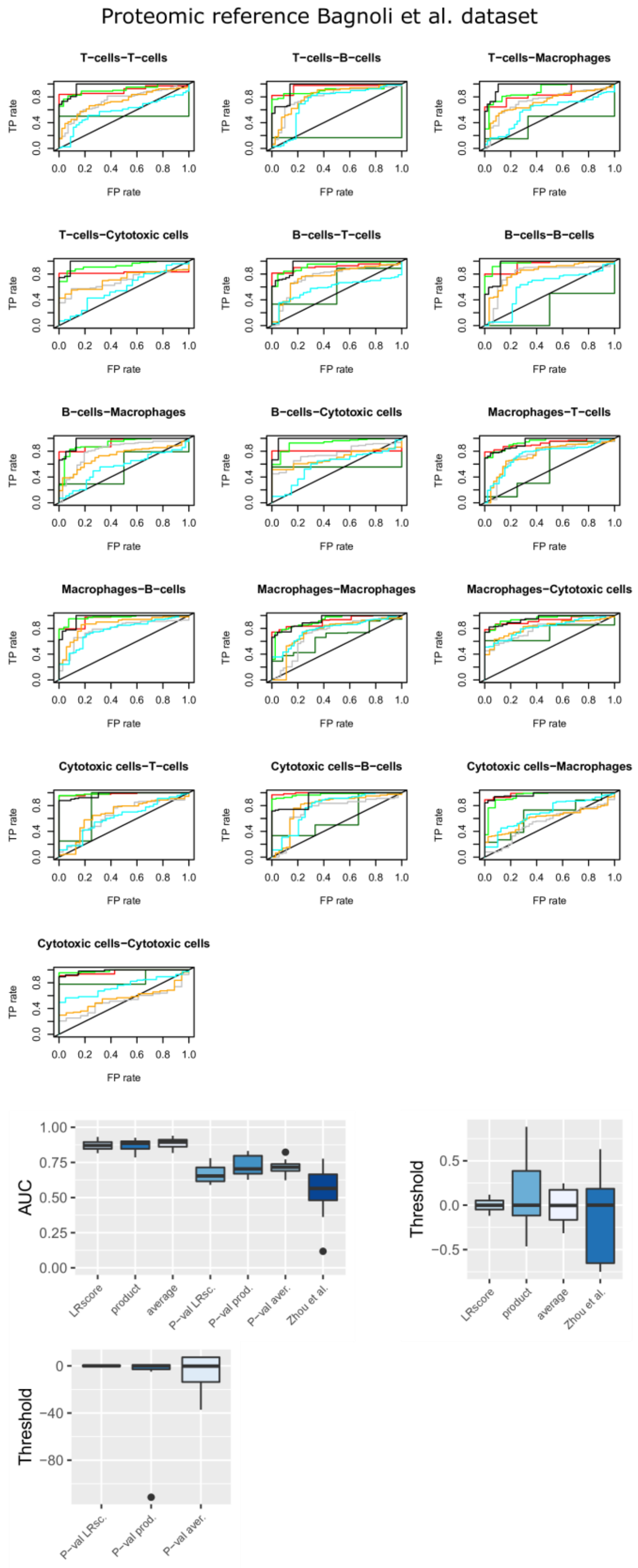

Supplementary Figure 8. ROC curves of LR pairs across cell populations of the PBMC data set (3) with respect to the proteomic reference (7).

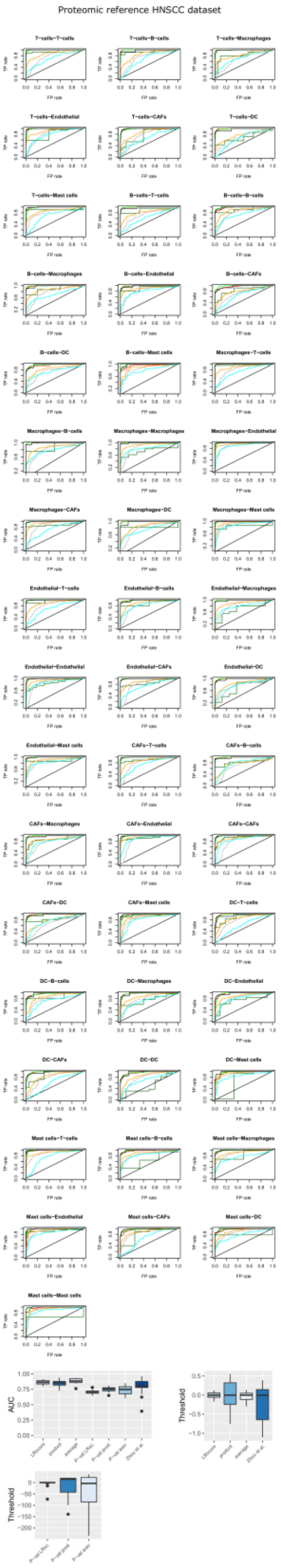

Supplementary Figure 9. ROC curves of LR pairs across cell populations of the HNSCC data set (4) with respect to the transcriptomic reference (7).

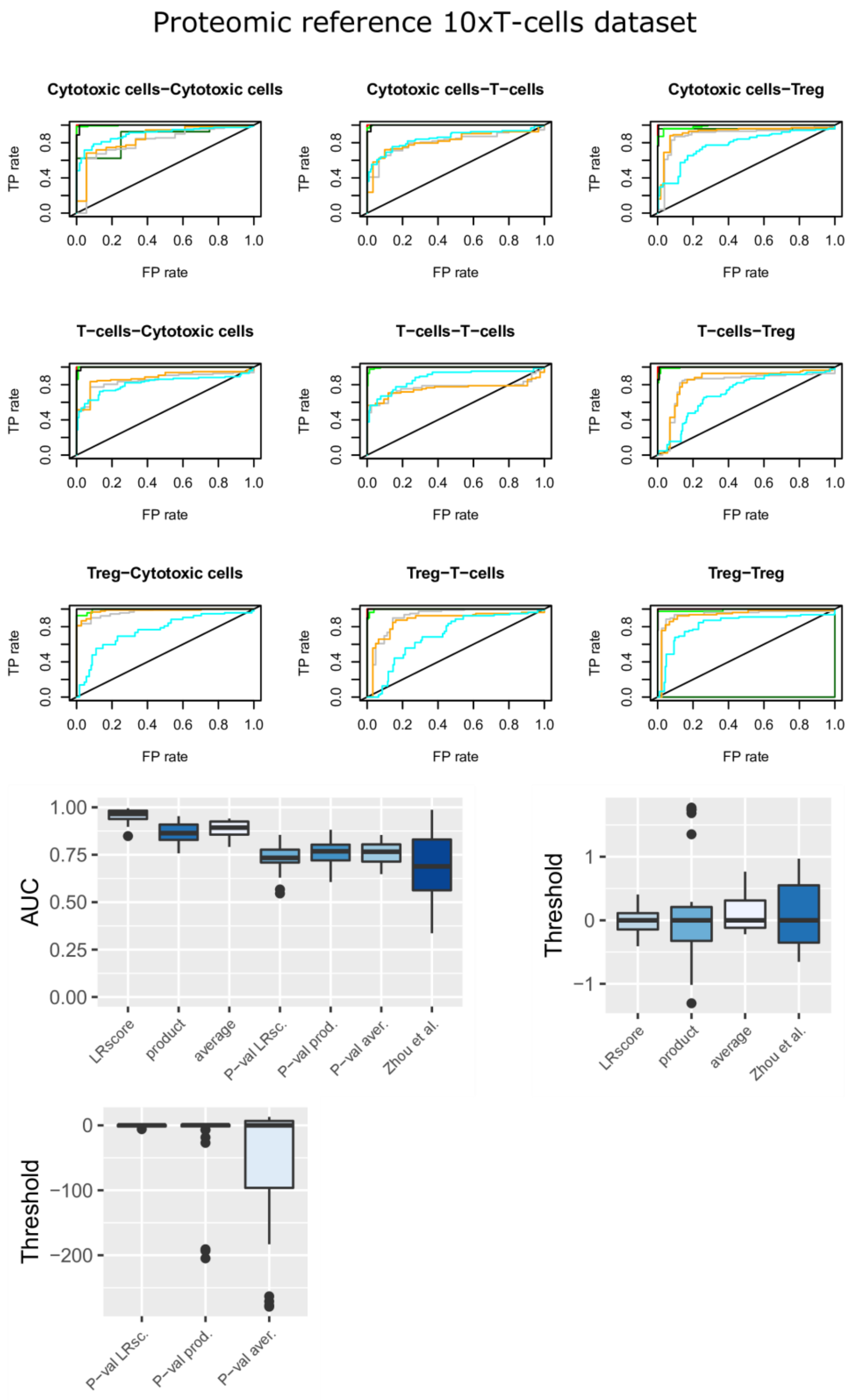

Supplementary Figure 10. ROC curves of LR pairs across cell populations of the 10xT data set (5) with respect to the transcriptomic reference (2).

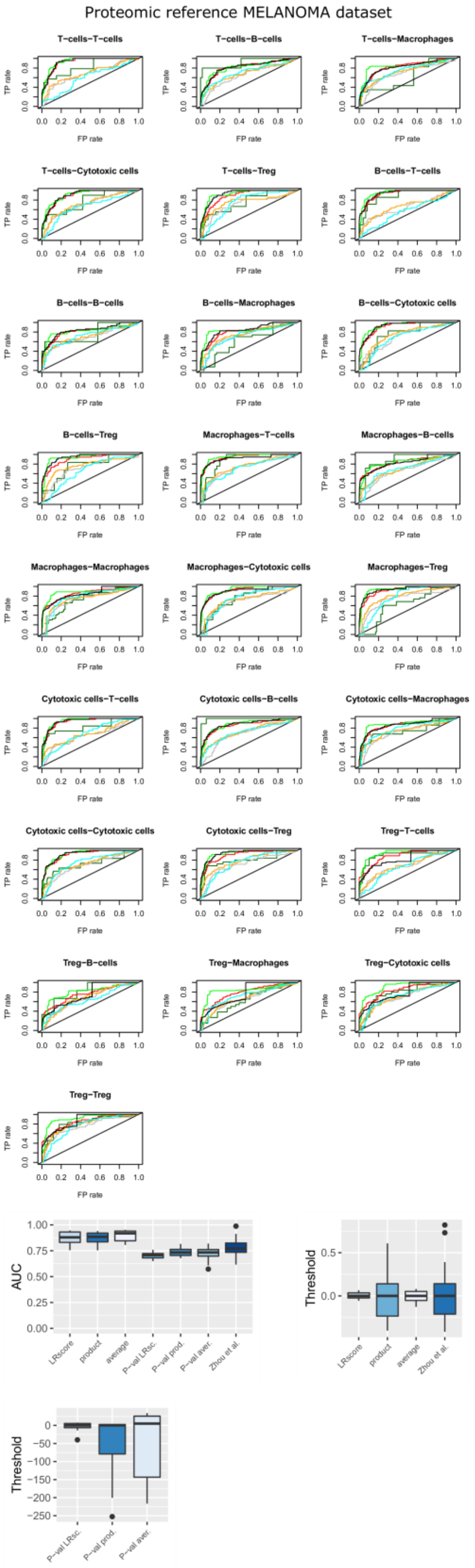

Supplementary Figure 11. ROC curves of LR pairs across cell populations of the MELANOMA data set (6) with respect to the proteomic reference (7).

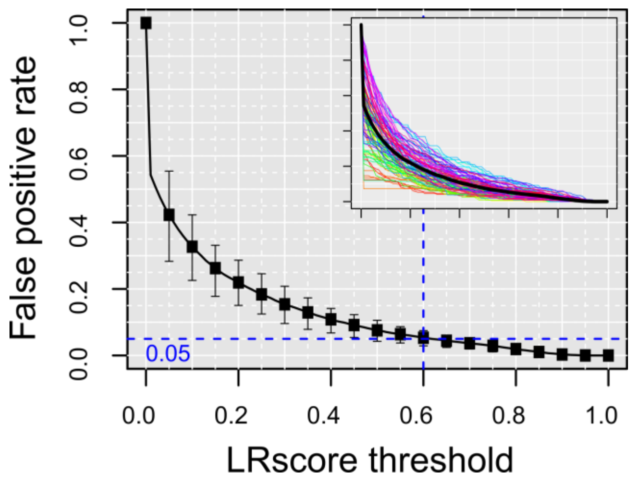

Supplementary Figure 12. Selection of the LRscore threshold requiring maximum 5% FP rate against the proteomic reference data set (7) for at least 75% of the ROC curves. Compare with Fig. 2D in the main text.

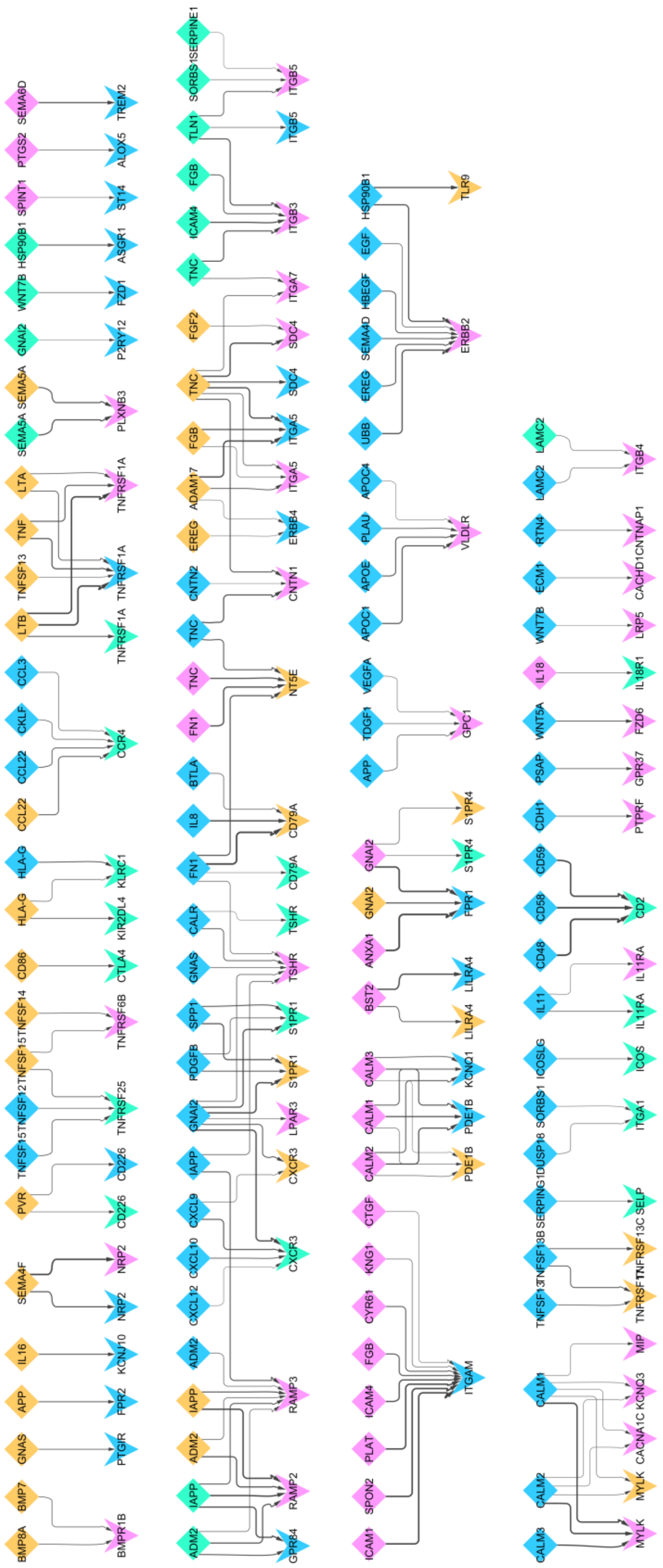

Supplementary Figure 13. Metastatic melanoma intercellular network, MELANOMA data set (6), patient 89.

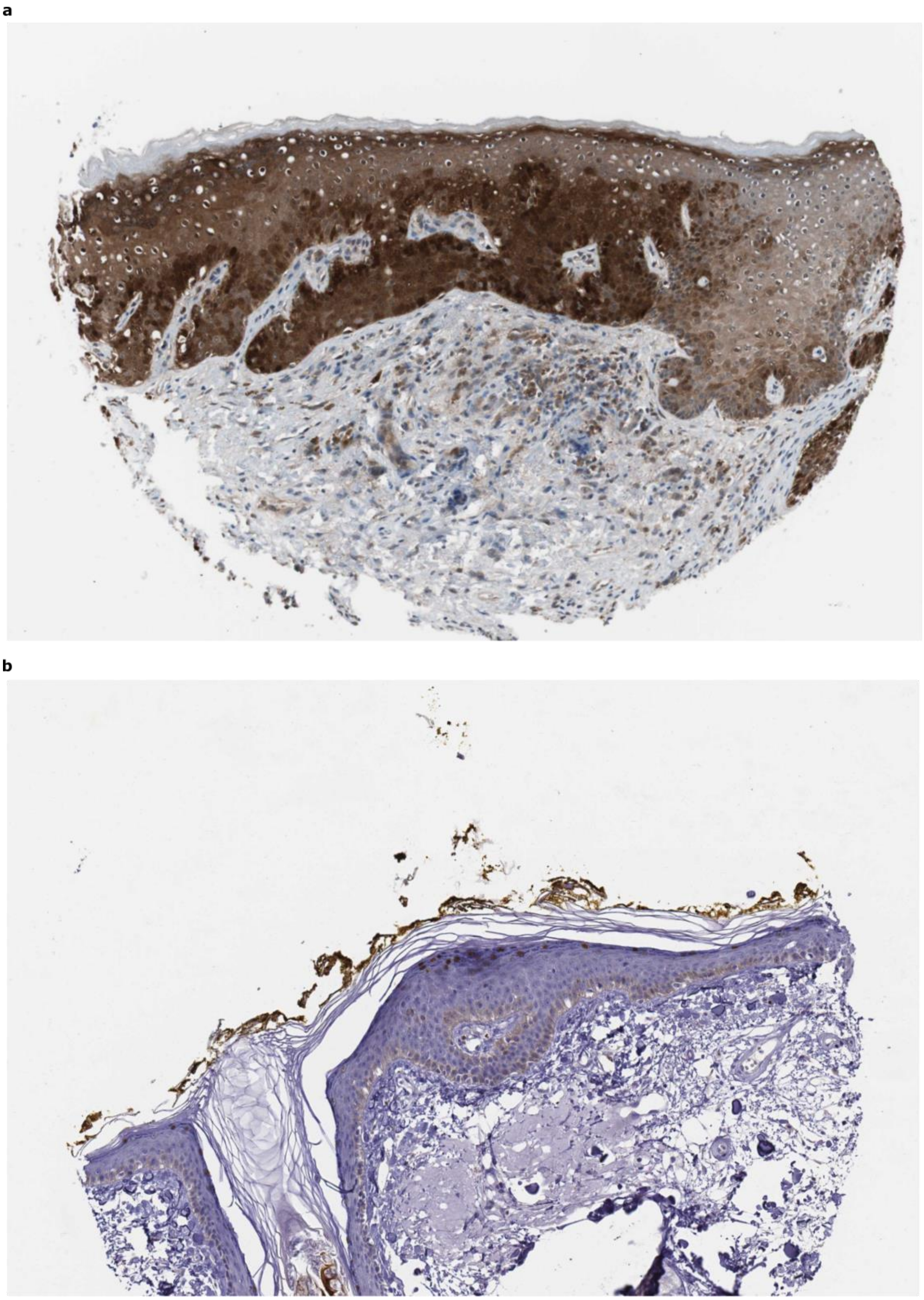

Supplementary Figure 14. Human Protein Atlas epidermis staining. (**a**) IL18 immunostaining in epidermal cells shows high expression in keratinocytes (CAB007772 andtibody). (**b**) PRDM1 immunostaining in epidermal cells shows medium expression in keratinocytes (HPA030033 andtibody).

Supplementary Table 1. Confirmation of mouse keratinized and suprabasal layer interactions. L/R in HPA indicate presence status in Human Protein Atlas skin data of the ligand, respectively the receptor (NA stands for absent, TRUE stands for present in the correct layer, FALSE for present but in the wrong layer). HPA is the HPA confirmation status. IF indicates the result of our IF mouse validation experiment. Literature indicates evidence from the literature. Confirmed is the final status. We considered that mouse IF or literature could supersede HPA. Low expression in HPA was considered positive. All HPA pictures are in supplementary data.

Keratinised	Suprabasal	LRscore	L in HPA	R in HPA	HPA	IF	Literature	Confirmed
Cdh1	Ptprf	0.905	TRUE	TRUE	yes	-	-	yes
Hbegf	Cd9	0.903	FALSE	TRUE	no	-	yes	yes
Calm1	Ptpra	0.880	TRUE	TRUE	yes	-	-	yes
Calm1	Egfr	0.862	TRUE	TRUE	yes	-	-	yes
Ubc	Ldlr	0.857	TRUE	TRUE	yes	-	-	yes
Ereg	Erbb2	0.855	NA	TRUE	NA	-	-	NA
Efnb2	Ephb6	0.852	TRUE	NA	NA	-	-	NA
Calm2	Egfr	0.851	TRUE	TRUE	yes	-	-	yes
Dsc3	Dsg1b	0.851	TRUE	NA	NA	-	-	NA
Ubc	Erbb2	0.849	TRUE	TRUE	yes	-	-	yes
Ubc	Adrb2	0.846	TRUE	NA	NA	-	-	NA
Btc	Erbb2	0.843	NA	TRUE	NA	-	-	NA
Rps27a	Ldlr	0.843	TRUE	TRUE	yes	-	-	yes
B2m	Tfrc	0.842	TRUE	TRUE	yes	-	-	yes
Apoe	Ldlr	0.840	TRUE	TRUE	yes	-	-	yes
Calm1	Insr	0.838	TRUE	TRUE	yes	-	-	yes
Anxa1	Egfr	0.837	TRUE	TRUE	yes	-	-	yes
Ptn	Sdc1	0.833	FALSE	TRUE	no	-	-	no
Rps27a	Erbb2	0.833	TRUE	TRUE	yes	-	-	yes
Rps27a	Adrb2	0.830	TRUE	NA	NA	-	-	NA
Cgn	F11r	0.829	TRUE	TRUE	yes	-	-	yes
Dsc1	Dsg1b	0.829	TRUE	NA	NA	-	-	NA
Hspa8	Ldlr	0.828	TRUE	TRUE	yes	-	-	yes
Calm2	Insr	0.826	TRUE	TRUE	yes	-	-	yes
Efna1	Ephb6	0.825	TRUE	NA	NA	-	-	NA
Ereg	Egfr	0.824	NA	TRUE	NA	-	-	NA
Efnb2	Epha4	0.822	TRUE	TRUE	yes	-	-	yes
Ubc	Fgfr2	0.822	TRUE	TRUE	yes	-	-	yes
Hsp90b1	Erbb2	0.819	TRUE	TRUE	yes	-	-	yes
Fabp5	Rxra	0.817	TRUE	TRUE	yes	-	-	yes
App	Ncstn	0.815	NA	TRUE	NA	-	-	NA
Hspa8	Adrb2	0.815	TRUE	NA	NA	-	-	NA
Ubb	Ldlr	0.813	TRUE	TRUE	yes	-	-	yes
App	Cav1	0.813	NA	TRUE	NA	-	-	NA
Actr2	Ldlr	0.812	TRUE	TRUE	yes	-	-	yes
Hbegf	**Cd44**	0.810	FALSE	TRUE	no	yes	yes	yes
Btc	Egfr	0.809	NA	TRUE	NA	-	-	NA
App	Lrp1	0.807	NA	TRUE	NA	-	-	NA
Hbegf	Cd82	0.804	FALSE	TRUE	no	-	yes	yes
Rps27a	Fgfr2	0.804	TRUE	TRUE	yes	-	-	yes
Ubb	Erbb2	0.802	TRUE	TRUE	yes	-	-	yes
Arpc5	Ldlr	0.799	NA	TRUE	NA	-	-	NA
**Psen1**	Notch1	0.799	FALSE	TRUE	no	yes	-	yes

Ubb	Adrb2	0.798	TRUE	NA	NA	-	-	NA
Dusp18	Itga6	0.798	TRUE	TRUE	yes	-	-	yes
Actr2	Adrb2	0.798	TRUE	NA	NA	-	-	NA
Efna5	Ephb6	0.797	TRUE	NA	NA	-	-	NA
Hbegf	Erbb2	0.792	FALSE	TRUE	no	-	yes	yes
Cgn	Ocln	0.792	TRUE	TRUE	yes	-	-	yes
Efna1	Epha4	0.790	TRUE	TRUE	yes	-	-	yes
Arpc5	Adrb2	0.784	NA	NA	NA	-	-	NA
Calm1	Abca1	0.784	TRUE	TRUE	yes	-	-	yes
Cdh1	Egfr	0.784	TRUE	TRUE	yes	-	-	yes
**Psen1**	**Cd44**	**0.780**	**FALSE**	**TRUE**	**no**	yes	-	yes
Adam17	Notch1	0.778	TRUE	TRUE	yes	-	-	yes
App	Gpc1	0.778	NA	TRUE	NA	-	-	NA
Apoe	Lrp1	0.776	TRUE	TRUE	yes	-	-	yes
Hsp90aa1	Fgfr3	0.776	NA	TRUE	NA	-	-	NA
Calr	Itgav	0.775	TRUE	TRUE	yes	-	-	yes
Cdh1	Igf1r	0.772	TRUE	TRUE	yes	-	-	yes
Hsp90aa1	Erbb2	0.771	NA	TRUE	NA	-	-	NA
Sorbs1	Itgb5	0.770	TRUE	TRUE	yes	-	-	yes
Efnb1	Ephb6	0.770	TRUE	NA	NA	-	-	NA
Ubb	Fgfr2	0.769	TRUE	TRUE	yes	-	-	yes
Calm2	Abca1	0.769	TRUE	TRUE	yes	-	-	yes
Pkm	**Cd44**	0.764	TRUE	TRUE	yes	yes	-	yes
Sema4a	Plxnb2	0.764	NA	TRUE	NA	-	-	NA
Calcb	Adrb2	0.762	NA	NA	NA	-	-	NA
Hsp90b1	Lrp1	0.762	TRUE	TRUE	yes	-	-	yes
Efna5	Epha4	0.759	TRUE	TRUE	yes	-	-	yes
Tgfa	Erbb2	0.759	TRUE	TRUE	yes	-	-	yes
Sptan1	Ptpra	0.758	FALSE	TRUE	no	-	-	no
Fst	Bmpr2	0.756	NA	FALSE	no	-	-	no
Arf1	Insr	0.755	TRUE	TRUE	yes	-	-	yes
Sptbn2	Ptpra	0.753	TRUE	TRUE	yes	-	-	yes
Lamb3	Col17a1	0.753	TRUE	TRUE	yes	-	-	yes
Hbegf	Egfr	0.751	FALSE	TRUE	no	-	yes	yes
Timp3	Adam17	0.750	NA	TRUE	NA	-	-	NA
Ecm1	Cachd1	0.750	TRUE	TRUE	yes	-	-	yes
**Psen1**	Notch2	0.750	FALSE	TRUE	no	yes	-	yes
Hspg2	Sdc1	0.742	TRUE	TRUE	yes	-	-	yes
Ubc	Ripk1	0.741	TRUE	TRUE	yes	-	-	yes
Adam10	Notch1	0.741	TRUE	TRUE	yes	-	-	yes
Calr	Lrp1	0.740	TRUE	TRUE	yes	-	-	yes
Ereg	Erbb3	0.734	NA	TRUE	NA	-	-	NA
Ptn	Plxnb2	0.732	NA	TRUE	NA	-	-	NA
Gas6	Tyro3	0.732	FALSE	TRUE	no	-	yes	yes
Dusp18	Cd151	0.728	TRUE	TRUE	yes	-	-	yes
Dusp18	Itgb4	0.728	TRUE	TRUE	yes	-	-	yes
Hsp90aa1	Egfr	0.727	NA	TRUE	NA	-	-	NA
Efnb2	Ephb3	0.721	TRUE	TRUE	yes	-	-	yes
Sorbs1	Insr	0.721	TRUE	TRUE	yes	-	-	yes
Efna1	Epha2	0.719	TRUE	NA	NA	-	-	NA
Rps27a	Ripk1	0.718	TRUE	TRUE	yes	-	-	yes
Rgmb	Bmpr2	0.716	NA	FALSE	no	-	-	no
Btc	Erbb3	0.715	NA	TRUE	NA	-	-	NA
Efna1	Epha1	0.714	TRUE	TRUE	yes	-	-	yes

Tgfa	Egfr	0.713	TRUE	TRUE	yes	-	-	yes
Efnb1	Erbb2	0.710	TRUE	TRUE	yes	-	-	yes
Lamc2	Col17a1	0.709	NA	TRUE	NA	-	-	NA
Mapk1	Fgfr2	0.708	TRUE	TRUE	yes	-	-	yes
Jag1	Notch1	0.705	TRUE	TRUE	yes	-	-	yes
Il18	Il18r1	0.704	TRUE	TRUE	yes	-	-	yes
**Psen1**	Ncstn	0.704	FALSE	TRUE	no	yes	-	yes
B2m	Cd3g	0.696	TRUE	TRUE	yes	-	-	yes
Spint1	St14	0.695	TRUE	NA	NA	-	-	NA
Ubc	Tgfbr1	0.692	TRUE	TRUE	yes	-	-	yes
Fgf18	Fgfr3	0.690	FALSE	TRUE	no	-	-	no
Hbegf	Prlr	0.689	FALSE	NA	no	-	yes	yes
Gpi1	Amfr	0.687	TRUE	TRUE	yes	-	-	yes
App	Tnfrsf21	0.685	NA	TRUE	NA	-	-	NA
Cdh1	Erbb3	0.682	TRUE	TRUE	yes	-	-	yes
Efna5	Epha2	0.681	TRUE	NA	NA	-	-	NA
Il1rn	Il1r2	0.677	TRUE	TRUE	yes	-	-	yes
Ubc	Tgfbr2	0.677	TRUE	NA	NA	-	-	NA
Bmp2	Bmpr2	0.676	NA	FALSE	no	-	-	no
Efna5	Epha1	0.676	TRUE	TRUE	yes	-	-	yes
Ubb	Ripk1	0.674	TRUE	TRUE	yes	-	-	yes
Thbs1	Sdc1	0.673	TRUE	TRUE	yes	-	-	yes
Apoe	Lrp5	0.670	TRUE	TRUE	yes	-	-	yes
Apoe	Sorl1	0.669	TRUE	TRUE	yes	-	-	yes
Rps27a	Tgfbr1	0.667	TRUE	TRUE	yes	-	-	yes
Efnb2	Rhbdl2	0.667	TRUE	TRUE	yes	-	-	yes
Gas6	Axl	0.666	FALSE	TRUE	no	-	yes	yes
Dusp18	Itgb1	0.666	TRUE	TRUE	yes	-	-	yes
Efnb2	Ephb4	0.666	TRUE	TRUE	yes	-	-	yes
Tgs1	Rxra	0.665	TRUE	TRUE	yes	-	-	yes
Gnai2	Egfr	0.664	TRUE	TRUE	yes	-	-	yes
Lama5	Sdc1	0.664	TRUE	TRUE	yes	-	-	yes
Bmp7	Bmpr2	0.662	NA	FALSE	no	-	-	no
Cgn	Tgfbr1	0.661	TRUE	TRUE	yes	-	-	yes
Areg	Egfr	0.659	TRUE	TRUE	yes	-	-	yes
Lrpap1	Ldlr	0.659	TRUE	TRUE	yes	-	-	yes
B2m	Hfe	0.654	TRUE	TRUE	yes	-	-	yes
Lpl	Sdc1	0.654	NA	TRUE	NA	-	-	NA
Cdh1	Lrp5	0.654	TRUE	TRUE	yes	-	-	yes
Ptdss1	Jmjd6	0.653	FALSE	TRUE	no	-	-	no
Sema4a	Plxnb1	0.652	NA	TRUE	NA	-	-	NA
Rps27a	Tgfbr2	0.651	TRUE	NA	NA	-	-	NA
Gnai2	Igf1r	0.649	TRUE	TRUE	yes	-	-	yes
Gnai2	Cav1	0.647	TRUE	TRUE	yes	-	-	yes
Lamb3	Itga6	0.646	TRUE	TRUE	yes	-	-	yes
Cgn	Tgfbr2	0.645	TRUE	NA	NA	-	-	NA
Agrn	Lrp4	0.644	TRUE	TRUE	yes	-	-	yes
Jag1	Notch2	0.643	TRUE	TRUE	yes	-	-	yes
Fgf18	Fgfr2	0.641	FALSE	TRUE	no	-	-	no
Psap	Sort1	0.638	TRUE	TRUE	yes	-	-	yes
Il1a	Il1r2	0.633	NA	TRUE	NA	-	-	NA
B2m	Cd247	0.631	TRUE	TRUE	yes	-	-	yes
Fgf22	Fgfr2	0.630	NA	TRUE	NA	-	-	NA
Gnai2	S1pr5	0.626	TRUE	TRUE	yes	-	-	yes

Psap	Celsr1	0.622	TRUE	NA	NA	-	-	NA
Il1rn	Il1r1	0.621	TRUE	TRUE	yes	-	-	yes
Ubb	Tgfbr1	0.619	TRUE	TRUE	yes	-	-	yes
Ltbp3	Itgb5	0.617	NA	TRUE	NA	-	-	NA
Tnf	Tnfrsf1a	0.617	NA	TRUE	NA	-	-	NA
Lin7c	Abca1	0.616	TRUE	TRUE	yes	-	-	yes
App	Cd74	0.614	NA	FALSE	no	-	-	no
Vim	**Cd44**	0.613	TRUE	TRUE	yes	-	-	yes
Adam9	Itga6	0.612	NA	TRUE	NA	-	-	NA
Col6a1	Itga6	0.611	TRUE	TRUE	yes	-	-	yes
Rgma	Bmpr2	0.610	TRUE	FALSE	no	-	-	no
Rtn4	Rtn4rl1	0.606	TRUE	TRUE	yes	-	-	yes
Calm1	Pde1b	0.606	TRUE	TRUE	yes	-	-	yes
Wnt7b	Fzd10	0.604	NA	TRUE	NA	-	-	NA
Lrpap1	Sort1	0.604	TRUE	TRUE	yes	-	-	yes
Ubb	Tgfbr2	0.602	TRUE	NA	NA	-	-	NA
Timp2	Itgb1	0.602	TRUE	TRUE	yes	-	-	yes
Efna4	Epha4	0.600	TRUE	TRUE	yes	-	-	yes
Efnb1	Ephb3	0.600	TRUE	TRUE	yes	-	-	yes
Epgn	Egfr	0.597	NA	TRUE	NA	-	-	NA
Ptn	Ptprs	0.597	FALSE	NA	no	-	-	no
Psap	Lrp1	0.596	TRUE	TRUE	yes	-	-	yes
Tgfa	Erbb3	0.595	TRUE	TRUE	yes	-	-	yes
Calr	Itga3	0.594	TRUE	TRUE	yes	-	-	yes
Adam17	Itgb1	0.594	TRUE	TRUE	yes	-	-	yes
Lamc2	Itga6	0.593	NA	TRUE	NA	-	-	NA
Tln1	Itgb5	0.588	TRUE	TRUE	yes	-	-	yes
Calm2	Pde1b	0.585	TRUE	TRUE	yes	-	-	yes
Pros1	Tyro3	0.585	TRUE	TRUE	yes	-	-	yes
Pthlh	Adrb2	0.584	TRUE	NA	NA	-	-	NA
Dsc3	Dsg2	0.584	TRUE	TRUE	yes	-	-	yes
Dusp18	Itga3	0.583	TRUE	TRUE	yes	-	-	yes
Bmp2	Bmpr1a	0.582	NA	TRUE	NA	-	-	NA
Adam10	Axl	0.579	TRUE	TRUE	yes	-	-	yes
Il1a	Il1r1	0.575	NA	TRUE	NA	-	-	NA
Arf1	Pld2	0.573	TRUE	TRUE	yes	-	-	yes
Rgmb	Neo1	0.571	NA	TRUE	NA	-	-	NA
B2m	Cd3d	0.570	TRUE	FALSE	no	-	-	no
Adam15	Itgav	0.569	FALSE	TRUE	no	-	-	no
Adam9	Itgav	0.567	NA	TRUE	NA	-	-	NA
Calm1	Mylk	0.567	TRUE	FALSE	no	-	-	no
Bmp7	Bmpr1a	0.567	NA	TRUE	NA	-	-	NA
Adam9	Itgb5	0.565	NA	TRUE	NA	-	-	NA
Col4a5	Cd47	0.564	NA	TRUE	NA	-	-	NA
Pdgfb	Itgav	0.562	TRUE	TRUE	yes	-	-	yes
Tnc	Sdc1	0.562	TRUE	TRUE	yes	-	-	yes
Lrpap1	Lrp1	0.560	TRUE	TRUE	yes	-	-	yes
Wnt3	Ryk	0.558	NA	TRUE	NA	-	-	NA
Dlk2	Notch1	0.557	TRUE	TRUE	yes	-	-	yes
Itgb3bp	Itgb5	0.554	TRUE	TRUE	yes	-	-	yes
Wnt7b	Fzd1	0.553	NA	TRUE	NA	-	-	NA
Rtn4	Gjb2	0.552	TRUE	TRUE	yes	-	-	yes
Lamb3	Cd151	0.552	TRUE	TRUE	yes	-	-	yes
Lamb3	Itgb4	0.552	TRUE	TRUE	yes	-	-	yes

Agrn	Lrp1	0.551	TRUE	TRUE	yes	-	-	yes
Vcl	Itgb5	0.549	TRUE	TRUE	yes	-	-	yes
Gnas	Adcy1	0.549	TRUE	TRUE	yes	-	-	yes
Calm2	Mylk	0.546	TRUE	FALSE	no	-	-	no
Calm3	Egfr	0.545	TRUE	TRUE	yes	-	-	yes
Dsc1	Dsg2	0.544	TRUE	TRUE	yes	-	-	yes
Bmp7	Acvr2a	0.540	NA	TRUE	NA	-	-	NA
App	Slc45a3	0.539	NA	TRUE	NA	-	-	NA
Sema6a	Plxna2	0.538	TRUE	TRUE	yes	-	-	yes
Efnb1	Ephb4	0.536	TRUE	TRUE	yes	-	-	yes
Ubc	Smad3	0.536	TRUE	TRUE	yes	-	-	yes
Gstp1	Traf2	0.536	TRUE	TRUE	yes	-	-	yes
Rtn4	Rtn4r	0.535	TRUE	NA	NA	-	-	NA
Areg	Erbb3	0.533	TRUE	TRUE	yes	-	-	yes
Inhbb	Acvr2a	0.532	NA	TRUE	NA	-	-	NA
Calm1	Hmmr	0.529	TRUE	TRUE	yes	-	-	yes
Col18a1	Itgb5	0.526	TRUE	TRUE	yes	-	-	yes
Il1a	Il1rap	0.526	NA	TRUE	NA	-	-	NA
Il18	Il1rl2	0.526	TRUE	TRUE	yes	-	-	yes
Col4a5	Itgav	0.526	NA	TRUE	NA	-	-	NA
Egf	Ldlr	0.522	NA	TRUE	NA	-	-	NA
B2m	Klrd1	0.521	TRUE	TRUE	yes	-	-	yes
Wnt7b	Lrp5	0.521	NA	TRUE	NA	-	-	NA
Thbs1	Itga6	0.520	TRUE	TRUE	yes	-	-	yes
Liph	Lpar2	0.519	TRUE	TRUE	yes	-	-	yes
Lpl	**Cd44**	0.518	NA	TRUE	NA	yes	-	NA
Sfrp1	Fzd6	0.515	TRUE	TRUE	yes	-	-	yes
Pdgfb	Lrp1	0.514	TRUE	TRUE	yes	-	-	yes
Timp2	Itga3	0.514	TRUE	TRUE	yes	-	-	yes
Thbs1	Cd47	0.512	TRUE	TRUE	yes	-	-	yes
Lama5	Itga6	0.509	TRUE	TRUE	yes	-	-	yes
Hspg2	Lrp1	0.509	TRUE	TRUE	yes	-	-	yes
Thbs2	Itga6	0.509	TRUE	TRUE	yes	-	-	yes
Fgf18	Fgfr1	0.508	FALSE	TRUE	no	-	-	no
Pros1	Axl	0.508	TRUE	TRUE	yes	-	-	yes
Ltbp1	Itgb5	0.508	TRUE	TRUE	yes	-	-	yes
Nampt	Insr	0.507	TRUE	TRUE	yes	-	-	yes
Rps27a	Smad3	0.507	TRUE	TRUE	yes	-	-	yes
Egf	Erbb2	0.505	NA	TRUE	NA	-	-	NA
Efna4	Epha2	0.504	TRUE	NA	NA	-	-	NA
Il1f6	Il1f5	0.502	NA	NA	NA	-	-	NA
Scgb1a1	Lmbr1l	0.502	FALSE	NA	no	-	-	no
Thbs2	Cd47	0.501	TRUE	TRUE	yes	-	-	yes

Supplementary Table 2. scTensor comparison results.

scTensor		SingleCellSignalR	
ligands	receptors	ligands	receptors
LGALS1	S100A6	PTN	PTPRZ1
CXCL10	IFITM3	LGALS1	CD69
DCN	PDGFRA		
COL3A1	PDGFRA		
COL1A1	PDGFRA		
COL1A1	ENG		
DCN	PDGFRB		
CXCL10	GNG11		
DCN	GPC3		
COL3A1	PDGFRB		
COL1A1	PDGFRB		
IGFBP5	GPC3		
SPARCL1	GPC3		
COL1A2	PDGFRA		

Supplementary Table 3. Paracrine interactions, comparison with PyMINEr on 10xPBMC data. SingleCellSignalR LRscore > 0.5.

	Number of inferred LR interactions		
**Cell types**	**SCSignalR**	**PyMINEr**	**Shared**
T-cells-B-cells	10	211	0
T-cells-Macrophages	17	669	0
T-cells-Cytotoxic cells	15	378	2
T-cells-Neutrophils	24	269	3
B-cells-T-cells	10	211	1
B-cells-Macrophages	28	721	0
B-cells-Cytotoxic cells	22	294	0
B-cells-Neutrophils	27	286	0
Macrophages-T-cells	17	669	1
Macrophages-B-cells	16	721	2
Macrophages-Cytotoxic cells	33	983	6
Macrophages-Neutrophils	9	1055	1
Cytotoxic cells-T-cells	19	378	0
Cytotoxic cells-B-cells	28	294	0
Cytotoxic cells-Macrophages	64	983	2
Cytotoxic cells-Neutrophils	50	399	5
Neutrophils-T-cells	38	269	2
Neutrophils-B-cells	42	286	4
Neutrophils-Macrophages	43	1055	3
Neutrophils-Cytotoxic cells	56	399	4

Supplementary Table 4. Paracrine and autocrine interactions, comparison with PyMINEr on 10xPBMC data. SingleCellSignalR LRscore > 0.5.

	Number of inferred LR interactions

Cell types	SCSignalR	PyMINEr	Shared
T-cells-T-cells	136	358	6
T-cells-B-cells	138	211	5
T-cells-Macrophages	202	669	7
T-cells-Cytotoxic cells	158	378	13
T-cells-Neutrophils	166	269	7
B-cells-T-cells	128	211	4
B-cells-B-cells	127	352	3
B-cells-Macrophages	193	721	5
B-cells-Cytotoxic cells	149	294	3
B-cells-Neutrophils	158	286	3
Macrophages-T-cells	179	669	12
Macrophages-B-cells	170	721	7
Macrophages-Macrophages	245	2800	41
Macrophages-Cytotoxic cells	189	983	17
Macrophages-Neutrophils	209	1055	19
Cytotoxic cells-T-cells	150	378	7
Cytotoxic cells-B-cells	148	294	6
Cytotoxic cells-Macrophages	216	983	15
Cytotoxic cells-Cytotoxic cells	165	512	17
Cytotoxic cells-Neutrophils	175	399	10
Neutrophils-T-cells	158	269	5
Neutrophils-B-cells	143	286	5
Neutrophils-Macrophages	228	1055	21
Neutrophils-Cytotoxic cells	179	399	8
Neutrophils-Neutrophils	195	384	9

Supplementary Table 5. Paracrine and autocrine interactions, comparison with CellPhoneDB on the 10xPBMC data. SingleCellSignalR LRscore > 0.5. Since CellPhoneDB returns its predictions without directionality, we put our results in the same format and each combination of cell types appears once only.

	**Number of inferred LR interactions**		
**Cell types**	**SCSignalR**	**CellPhoneDB**	**Shared**
T-cells-T-cells	110	6	4
T-cells-B-cells	158	15	10
T-cells-Macrophages	224	28	15
T-cells-Cytotoxic cells	156	15	8
T-cells-Neutrophils	219	26	13
T-cells-Treg	147	8	6
B-cells-B-cells	125	8	3
B-cells-Macrophages	234	32	14
B-cells-Cytotoxic cells	186	20	8
B-cells-Neutrophils	229	30	14
B-cells-Treg	168	17	10
Macrophages-Macrophages	225	34	14
Macrophages-Cytotoxic cells	269	41	20
Macrophages-Neutrophils	242	37	17
Macrophages-Treg	234	34	16
Cytotoxic cells-Cytotoxic cells	153	15	6
Cytotoxic cells-Neutrophils	252	37	23
Cytotoxic cells-Treg	182	14	9
Neutrophils-Neutrophils	209	28	16
Neutrophils-Treg	227	30	14
Treg-Treg	111	4	3

Supplementary Table 6. Paracrine and autocrine interactions, comparison with iTALK on 10xPBMC data. 10xPBMC dada taken from iTALK GitHub for this particular case to match iTALK manuscript. SingleCellSignalR LRscore > 0.5.

	Number of inferred LR interactions		
**Cell types**	**SCSignalR**	**iTALK**	**Shared**
cd56_nk-cd14_monocytes	134	118	97
cd56_nk-b_cells	100	118	68
cd56_nk-cytotoxic_t	140	118	100
cd56_nk-regulatory_t	122	118	88
cd56_nk-memory_t	122	118	94
cd56_nk-naive_t	93	118	68
cd14_monocytes-cd56_nk	157	118	105
cd14_monocytes-b_cells	107	118	73
cd14_monocytes-cytotoxic_t	137	118	97
cd14_monocytes-regulatory_t	120	118	88
cd14_monocytes-memory_t	128	118	99
cd14_monocytes-naive_t	97	118	71
b_cells-cd56_nk	141	118	89
b_cells-cd14_monocytes	119	118	83
b_cells-cytotoxic_t	124	118	84
b_cells-regulatory_t	119	118	86
b_cells-memory_t	111	118	85
b_cells-naive_t	88	118	66
cytotoxic_t-cd56_nk	148	118	98
cytotoxic_t-cd14_monocytes	114	118	80
cytotoxic_t-b_cells	88	118	56
cytotoxic_t-regulatory_t	106	118	76
cytotoxic_t-memory_t	107	118	81
cytotoxic_t-naive_t	81	118	58
regulatory_t-cd56_nk	150	118	99
regulatory_t-cd14_monocytes	125	118	93
regulatory_t-b_cells	99	118	66
regulatory_t-cytotoxic_t	136	118	96
regulatory_t-memory_t	114	118	86
regulatory_t-naive_t	89	118	65
memory_t-cd56_nk	160	118	106
memory_t-cd14_monocytes	130	118	95
memory_t-b_cells	106	118	70
memory_t-cytotoxic_t	136	118	94
memory_t-regulatory_t	116	118	81
memory_t-naive_t	94	118	68
naive_t-cd56_nk	144	118	94
naive_t-cd14_monocytes	119	118	86
naive_t-b_cells	92	118	61
naive_t-cytotoxic_t	126	118	87
naive_t-regulatory_t	106	118	74
naive_t-memory_t	103	118	79

Supplementary Box 1. Example of SingleCellSIgnalR usage.

```
library(SingleCellSignalR)

# Define your working directory
setwd("~/example/")

# Define the file of interest you want to work with
file = "example_dataset.txt"

# Prepare the data for the analysis
data = data_prepare(file = file)
genes = rownames(data)

# Proceed to clustering
clust = clustering(data = data,n = 10, method = "simlr")
cluster = clust$cluster
tsne = clust$`t-SNE`

# Cell classification
my.markers = markers(c("immune"))
class = cell_classifier(data=data, genes=genes, markers = my.markers)

# Cluster analysis
clust.ana = cluster_analysis(data = data, genes = genes, cluster = cluster, markers
= my.markers)

# Proceed to cell signaling
signal = cell_signaling(data = data, genes = genes, cluster = cluster,species =
"homo sapiens")

# Visualization
visualize(inter = signal)
visualize(inter = signal, show.in = c(5))

expression.plot(data = data, name = "CD14", tsne = tsne)
expression.plot.2(data = data, name.1 = "CD40LG", name.2 = "CD40", tsne = tsne)

# Create interface network
inter.net = inter_network(data = data, signal = signal, genes = genes, cluster =
cluster)

# Show interactions downstream a specific receptor
intra = intra_network(goi = "S1PR1",data = data,genes = genes,cluster =
cluster,coi="cluster 3",signal=signal)
```

Supplementary Box 2. Example of integration with an external tool (Seurat).

```
library(SingleCellSignalR)
library(Seurat)

# Define your working directory
setwd("~/example/")

# Pre-processing using Seurat (https://satijalab.org/seurat/)
pbmc.data <- Read10X(data.dir = "./filtered_feature_bc_matrix/")
pbmc <- CreateSeuratObject(counts = pbmc.data, project = "pbmc1k")

# Data filtering and normalization
pbmc[["percent.mt"]] <- PercentageFeatureSet(pbmc, pattern = "^MT-")
pbmc <- subset(pbmc, subset = nFeature_RNA > 40)
pbmc <- NormalizeData(pbmc,scale.factor = 10000)
pbmc <- FindVariableFeatures(pbmc, selection.method = "vst", nfeatures = 2000)

# Data clustering
all.genes <- rownames(pbmc)
pbmc <- ScaleData(pbmc, features = all.genes)

pbmc <- RunPCA(pbmc, features = VariableFeatures(object = pbmc))
pbmc <- FindNeighbors(pbmc, dims = 1:10)
pbmc <- FindClusters(pbmc, resolution = 0.1)

# Retreiving the results of the preprocessing from the Seurat object
cluster = as.numeric(Idents(pbmc))
data = data.frame(pbmc[["RNA"]]@data)

# Ligand/Receptor analysis using SingleCellSignalR
signal = cell_signaling(data=data,genes=all.genes,cluster=cluster)

# Visualization
visualize(signal)
intra = intra_network("S1PR1",data,all.genes,cluster,"cluster 3",signal = signal)
```

Supplementary Methods

Built-in call type calling algorithm

In case SingleCellSignalR users do not want to use a specialized tool to infer the cell types corresponding to the cell clusters, we implemented the following algorithm.

A limited number of curated gene signatures are integrated with SingleCellSignalR that cover common cell types for our laboratory applications:

- Immune: T cells, B cells, macrophages, cytotoxic cells, dendritic cells, mast cells, neutrophils, natural killer cells, regulatory T cells;
- Tumor microenvironment: endothelial cells, cancer-associated fibroblasts;
- Melanoma: melanoma cancer cells;
- Breast cancer: triple-negative, HER+, and ER+ breast cancer cells.

They are stored in a format identical to PanglaoDB (9) exports such that users can easily add cell types from this rich source or provide their own. A cell type t signature is comprised of genes $g_{t,1}, \cdots, g_{t,k_t}$ and its average expression in each cell j is $a_{t,j} = \frac{1}{k_t}\sum_{i=1}^{k_t} c_{\mathrm{row}(g_{t,i}),j}$, with $\mathrm{row}(g_{t,i})$ the row in C representing gene $g_{t,i}$. C is the preprocessed gene expression matrix, i.e., the normalized and clustered read counts. A matrix $A = (a_{t,j})$ is obtained with the average expression of all the signatures, which we normalize imposing that columns sum to 1. The normalized matrix is called $\tilde{A}$. We adjust a threshold a^* such that the condition $\tilde{a}_{t,j} > a^*$ maximizes the number of cells assigned to a single cell type.

We illustrate the application of the algorithm above to 10xPBMC data (1) comprised of 7,857 cells. After data normalization with our default procedure (Materials and Methods), SIMLR applied with default parameters identified 6 cell clusters (**Suppl. Fig. 15A**). Using the predefined “immune” gene signatures revealed the predominant subpopulations (>10 cells). Two SIMLR clusters were homogeneous (B cells in cluster 1 and cytotoxic cells in cluster 6), while the other 4 clusters were mixed (**Suppl. Fig. 15B**). At the individual transcriptome level, cell type calling was unambiguous for 94% of the cells (**Suppl. Fig. 15C**). This classification was projected in the t-SNE coordinates (**Suppl. Fig. 15D**). Less than 2% of cells were assigned to more than one type. They corresponded to lymphoid and myeloid cells in an intermediate state with a dual assignment: T cells and cytotoxic T cells, or macrophages and neutrophils. In the remaining 4%, SingleCellSignalR cell type caller failed to attribute any cell type. Cells assigned to multiple or no cell type are featured as gray dots in **Suppl. Fig. 15D**. Cluster 2 was refined by cell type calling to unravel a gradient from neutrophils to macrophages, which come from the same lineage. Similarly, cluster 4 featured a gradient from cytotoxic cells to T cells, with a small number of regulatory T in the middle. Cluster 5 was made of B cells in a different transcriptomic state compared to cluster 1. The large cluster 4 that we found to cover T cells and cytotoxic cells could be further decomposed in multiple cytotoxic subpopulations. No NK cells were found in clusters 4 and 6.

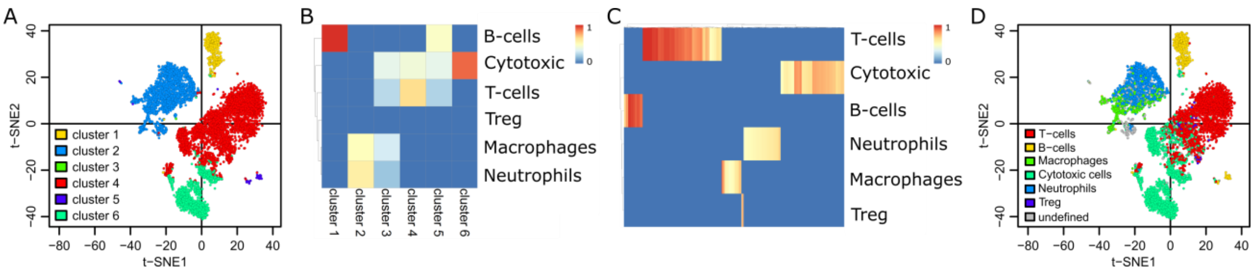

Supplementary Figure 15. Illustration of cell type calling algorithm.

A second illustration was obtained re-classifying the cells of the MELANOMA data set (6) and comparing with the authors original calling. We obtained very similar results as shown in **Suppl. Table 7** below.

Supplementary Table 7. Comparison of cell type calling between original MELANOMA data set values (patients 79, 80, 88, and 89) and SingleCellSignalR algorithm predictions.

	T-cells	B-cells	Macrophages	Endothelial cells	CAFs	NK cells	Melanoma
**Mel79**	304	79	0	2	1	1	468
**Mel79 - SingleCellSignalR**	293	82	0	2	2	0	500
**Mel80**	212	49	0	29	23	4	125
**Mel80 - SingleCellSignalR**	224	55	0	30	29		126
**Mel88**	112	16	41	0	2	9	112
**Mel88 - SingleCellSignalR**	117	22	46	0	0	0	134
**Mel89**	201	106	26	1	0	1	98
**Mel89 - SingleCellSignalR**	218	113	30	0	0	0	107

Autocrine *versus* paracrine interactions

By default, an interaction between two cell types A and B, with ligand in A and receptor in B, is considered paracrine if the A cells do not express the receptor (and *vice versa*). This cutoff can be raised, e.g., to 2%, to accommodate slight expression of the receptor in A (or the ligand in B) and maintain the paracrine classification. In this work, we used 0% everywhere but for the mouse interfollicular epidermis data (2%). In every case, all the LR pairs considered significant (LR score above threshold) will be classified as paracrine or autocrine.

References

1. 8k PBMCs from a Healthy Donor (2017).

2. Ramilowski,J.A., Goldberg,T., Harshbarger,J., Kloppmann,E., Lizio,M., Satagopam,V.P., Itoh,M., Kawaji,H., Carninci,P., Rost,B., *et al.* (2015) A draft network of ligand–receptor-mediated multicellular signalling in human. *Nature Communications*, **6**, 7866.

3. Bagnoli,J.W., Ziegenhain,C., Janjic,A., Wange,L.E., Vieth,B., Parekh,S., Geuder,J., Hellmann,I. and Enard,W. (2018) Sensitive and powerful single-cell RNA sequencing using mcSCRB-seq. *Nat Commun*, **9**, 2937.

4. Puram,S.V., Tirosh,I., Parikh,A.S., Patel,A.P., Yizhak,K., Gillespie,S., Rodman,C., Luo,C.L., Mroz,E.A., Emerick,K.S., *et al.* (2017) Single-Cell Transcriptomic Analysis of Primary and Metastatic Tumor Ecosystems in Head and Neck Cancer. *Cell*, **171**, 1611-1624.e24.

5. 4k Pan T Cells from a Healthy Donor (2017).

6. Tirosh,I., Izar,B., Prakadan,S.M., Wadsworth,M.H., Treacy,D., Trombetta,J.J., Rotem,A., Rodman,C., Lian,C., Murphy,G., *et al.* (2016) Dissecting the multicellular ecosystem of metastatic melanoma by single-cell RNA-seq. *Science*, **352**, 189–196.

7. Rieckmann,J.C., Geiger,R., Hornburg,D., Wolf,T., Kveler,K., Jarrossay,D., Sallusto,F., Shen-Orr,S.S., Lanzavecchia,A., Mann,M., *et al.* (2017) Social network architecture of human immune cells unveiled by quantitative proteomics. *Nat. Immunol.*, **18**, 583–593.

8. Tyler,S.R., Rotti,P.G., Sun,X., Yi,Y., Xie,W., Winter,M.C., Flamme-Wiese,M.J., Tucker,B.A., Mullins,R.F., Norris,A.W., *et al.* (2019) PyMINEr Finds Gene and Autocrine-Paracrine Networks from Human Islet scRNA-Seq. *Cell Rep*, **26**, 1951-1964.e8.

9. Franzén,O., Gan,L.-M. and Björkegren,J.L.M. (2019) PanglaoDB: a web server for exploration of mouse and human single-cell RNA sequencing data. *Database (Oxford)*, **2019**.
